# Supplementary material for: Think then act or act then think?
Source: PLoS One. 2018 Nov 14;13(11):e0206166. doi: 10.1371/journal.pone.0206166 (PMC6235276; doi:10.1371/journal.pone.0206166)
Supplement: S1 Appendix — (PDF) [file pone.0206166.s001.pdf]

Think then act or act then think?

Arkadiusz Jędrzejewski, Grzegorz Marcjasz, Paul R. Nail, Katarzyna Sznajd-Weron

Supporting information: S1 Appendix.

Derivation of formulas for the critical points and stationary states.

In the main text, we include an expression for the critical point p^* that separates low- and high-independence phases in the case of continuous phase transitions and becomes the lower bound of a metastable region in the case of discontinuous phase transitions. Here, we show how to derive the formula for this specific point. Presented method is quite general and can be applied to other similar models. First, let us recall the transition rates for the concentrations of positive public opinions

$$\gamma_S^+ = (1-c_S)\left\{pc_\sigma + (1-p)\left[c_\sigma(1-(1-c_S)^q) + (1-c_\sigma)c_S^q\right]\right\}, \tag{1}$$

$$\gamma_S^- = c_S\left\{p(1-c_\sigma) + (1-p)\left[c_\sigma(1-c_S)^q + (1-c_\sigma)(1-c_S^q)\right]\right\}, \tag{2}$$

and private ones

$$\gamma_\sigma^+ = (1-c_\sigma)\left[p/2 + (1-p)c_S^q\right], \tag{3}$$

$$\gamma_\sigma^- = c_\sigma\left[p/2 + (1-p)(1-c_S)^q\right]. \tag{4}$$

Now, for each opinion level, let us define a quantity that can be thought of as net force acting on our system, which can increase or decrease corresponding concentration of positive opinions:

$$F_S(c_S, c_\sigma, p) = \gamma_S^+ - \gamma_S^-, \tag{5}$$

$$F_\sigma(c_S, c_\sigma, p) = \gamma_\sigma^+ - \gamma_\sigma^-. \tag{6}$$

We treat q as a fixed parameter, so we do not include it in arguments of the above functions. Naturally, in the stationary state, our forces are equal to zero $F_S(c_S, c_\sigma, p) = 0$ and $F_\sigma(c_S, c_\sigma, p) = 0$. This creates two conditions for the stationary values of c_S and c_σ. Moreover, these stationary values of concentrations are coupled, and we can easily derive this dependency using the above vanishing force condition for the private opinion level together with Eqs. (3) and (4). This procedure leads to the expression for the stationary values of c_σ in the following form

$$c_\sigma = \frac{\frac{1}{2}p + (1-p)c_S^q}{p + (1-p)\left[c_S^q + (1-c_S)^q\right]}. \tag{7}$$

As we see, in the stationary state, $c_\sigma(c_S, p)$ depends only on c_S and p. Therefore, in this state, the force related to the public opinion level is, in fact, a two-variable function that equals to zero

$$F_S(c_S, p) = 0 \tag{8}$$

since we can eliminate c_σ from the formulas using Eq. (7). Note that the above expression is also an implicit equation for the stationary values of the concentration of positive opinions at the public level c_S where

$$F_S(c_S, p) = \frac{1}{2}\left[(1-c_S)^q - c_S^q\right]p^2 - \frac{1}{2}(1 - c_S^q - (1-c_S)^q)(2c_S - 1)p + (1-c_S)c_S^q - c_S(1-c_S)^q. \tag{9}$$

Now, differentiating Eq. (8) with respect to the concentration gives

$$\frac{\partial F_S(c_S, p)}{\partial c_S} + \frac{\partial F_S(c_S, p)}{\partial p}\frac{dp}{dc_S} = 0. \tag{10}$$

But since the critical point corresponds to the extremum of $p(c_S)$, the first derivative of the independence level over c_S vanishes at $(c_S, p) = (1/2, p^*)$, so that we have

$$\left.\frac{dp}{dc_S}\right|_{(1/2,p^*)} = 0. \tag{11}$$

Hence, evaluating Eq. (10) at the critical point gives the condition for its derivation

$$\left.\frac{\partial F_S(c_S, p)}{\partial c_S}\right|_{(1/2,p^*)} = 0. \tag{12}$$

Following the above instructions, one can arrive at the quadratic equation for the critical point

$$q(p^*)^2 + (2^{q-1} - 1)p^* - q + 1 = 0. \tag{13}$$

Finally, taking into account only a positive root, since $p^* > 0$, results in the following formula

$$p^* = \frac{1 - 2^{q-1} + \sqrt{(1 - 2^{q-1})^2 + 4q(q-1)}}{2q} \tag{14}$$

presented in the main text.
