# Supplementary material for: Think then act or act then think?
Source: PLoS One. 2018 Nov 14;13(11):e0206166. doi: 10.1371/journal.pone.0206166 (PMC6235276; doi:10.1371/journal.pone.0206166)
Supplement: S2 Appendix — (PDF) [file pone.0206166.s002.pdf]

Think then act or act then think?

Arkadiusz Jędrzejewski, Grzegorz Marcjasz, Paul R. Nail, Katarzyna Sznajd-Weron

Supporting information: S2 Appendix.

Transition rates in the exact mathematical formulations of the models.

Herein, we present expressions for all transition rates related to four concentrations $c_{\uparrow\uparrow}$, $c_{\uparrow\downarrow}$, $c_{\downarrow\downarrow}$, and $c_{\downarrow\uparrow}$ for both updating scheme orders of private and public opinions. These four quantities fully describe the models' behavior since based on them we can obtain the concentrations of positive opinions on public and private levels as well as the dissonance level using the following formulas mentioned already in the main text:

$$c_S = c_{\uparrow\uparrow} + c_{\uparrow\downarrow}, \tag{1}$$
$$c_\sigma = c_{\uparrow\uparrow} + c_{\downarrow\uparrow}, \tag{2}$$
$$d = c_{\uparrow\downarrow} + c_{\downarrow\uparrow}. \tag{3}$$

We recall that in our notation convention the first lower index, that is to say, the first arrow, corresponds to the public opinion state whereas the second one to the private opinion state. The arrow direction, $\uparrow$ or $\downarrow$, corresponds to two different opinion states, 1 or -1.

<u>Think then act (TA) model:</u>

$$\begin{aligned}\gamma^+_{\uparrow\uparrow} =&(c_{\uparrow\downarrow}+c_{\downarrow\downarrow})\left[\frac{p^2}{2}+\frac{p}{2}(1-p)(1-(1-c_S)^q)+p(1-p)c_S^q+(1-p)^2c_S^q(1-(1-c_S)^q)\right]\\ &+c_{\downarrow\uparrow}\left[\frac{p^2}{2}+\frac{3p}{2}(1-p)(1-(1-c_S)^q)+(1-p)^2(1-(1-c_S)^q)^2\right],\end{aligned} \tag{4}$$

$$\gamma^-_{\uparrow\uparrow} =c_{\uparrow\uparrow}\left[\frac{p}{2}(1-p)(1-c_S)^q+\frac{p}{2}+(1-p)(1-c_S)^q+(1-p)^2(1-(1-c_S)^q)(1-c_S)^q\right], \tag{5}$$

$$\gamma^+_{\uparrow\downarrow} =(c_{\uparrow\uparrow}+c_{\downarrow\uparrow})\left[\frac{p}{2}(1-p)c_S^q+(1-p)^2(1-c_S)^qc_S^q\right]+c_{\downarrow\downarrow}\left[\frac{p}{2}(1-p)c_S^q+(1-p)^2(1-c_S^q)c_S^q\right], \tag{6}$$

$$\gamma^-_{\uparrow\downarrow} =c_{\uparrow\downarrow}\left[\frac{p^2}{2}+\frac{3p}{2}(1-p)(1-c_S^q)+\frac{p}{2}+(1-p)c_S^q+(1-p)^2(1-c_S^q)^2\right], \tag{7}$$

$$\begin{aligned}\gamma^+_{\downarrow\downarrow} =&(c_{\uparrow\uparrow}+c_{\downarrow\uparrow})\left[\frac{p^2}{2}+\frac{p}{2}(1-p)(1-c_S^q)+p(1-p)(1-c_S)^q+(1-p)^2(1-c_S)^q(1-c_S^q)\right]\\ &+c_{\uparrow\downarrow}\left[\frac{p^2}{2}+\frac{3p}{2}(1-p)(1-c_S^q)+(1-p)^2(1-c_S^q)^2)\right],\end{aligned} \tag{8}$$

$$\gamma^-_{\downarrow\downarrow} =c_{\downarrow\downarrow}\left[\frac{p}{2}(1-p)c_S^q+\frac{p}{2}+(1-p)c_S^q+(1-p)^2(1-c_S^q)c_S^q\right], \tag{9}$$

$$\begin{aligned}\gamma^+_{\downarrow\uparrow} =&c_{\uparrow\uparrow}\left[\frac{p}{2}(1-p)(1-c_S)^q+(1-p)^2(1-(1-c_S)^q)(1-c_S)^q\right]\\ &+(c_{\uparrow\downarrow}+c_{\downarrow\downarrow})\left[\frac{p}{2}(1-p)(1-c_S)^q+(1-p)^2c_S^q(1-c_S)^q\right],\end{aligned} \tag{10}$$

$$\gamma^-_{\downarrow\uparrow} =c_{\downarrow\uparrow}\left[\frac{p^2}{2}+\frac{3p}{2}(1-p)(1-(1-c_S)^q)+\frac{p}{2}+(1-p)(1-c_S)^q+(1-p)^2(1-(1-c_S)^q)^2\right]. \tag{11}$$

Act then think (AT) model:

$$\gamma^{+}_{\uparrow\uparrow} = (c_{\uparrow\downarrow} + c_{\downarrow\downarrow}) \left[\frac{p}{2}(1-p)c_S^q + (1-p)^2 c_S^{2q}\right] + c_{\downarrow\uparrow}\left[\frac{p^2}{2} + \frac{3p}{2}(1-p)(1-(1-c_S)^q) + (1-p)^2(1-(1-c_S)^q)^2\right], \tag{12}$$

$$\gamma^{-}_{\uparrow\uparrow} = c_{\uparrow\uparrow}\left[\frac{p^2}{2} + p(1-p)(1-c_S)^q + (1-p)(1-c_S)^q + \frac{p}{2}(1-p)(1-(1-c_S)^q) + (1-p)^2(1-(1-c_S)^q)(1-c_S)^q\right], \tag{13}$$

$$\gamma^{+}_{\uparrow\downarrow} = (c_{\uparrow\uparrow} + c_{\downarrow\uparrow})\left[\frac{p^2}{2} + p(1-p)(1-c_S)^q + \frac{p}{2}(1-p)(1-(1-c_S)^q) + (1-p)^2(1-(1-c_S)^q)(1-c_S)^q\right] + c_{\downarrow\downarrow}\left[\frac{p}{2}(1-p)c_S^q + (1-p)^2 c_S^q(1-c_S^q)\right], \tag{14}$$

$$\gamma^{-}_{\uparrow\downarrow} = c_{\uparrow\downarrow}\left[p + (1-p)(1-c_S^q) + \frac{p}{2}(1-p)c_S^q + (1-p)^2 c_S^{2q}\right], \tag{15}$$

$$\gamma^{+}_{\downarrow\downarrow} = (c_{\uparrow\uparrow} + c_{\downarrow\uparrow})\left[\frac{p}{2}(1-p)(1-c_S)^q + (1-p)^2(1-c_S)^{2q}\right] + c_{\uparrow\downarrow}\left[\frac{p^2}{2} + \frac{3p}{2}(1-p)(1-c_S^q) + (1-p)^2(1-c_S^q)^2\right], \tag{16}$$

$$\gamma^{-}_{\downarrow\downarrow} = c_{\downarrow\downarrow}\left[\frac{p^2}{2} + p(1-p)c_S^q + (1-p)c_S^q + \frac{p}{2}(1-p)(1-c_S^q) + (1-p)^2(1-c_S^q)c_S^q\right], \tag{17}$$

$$\gamma^{+}_{\downarrow\uparrow} = c_{\uparrow\uparrow}\left[\frac{p}{2}(1-p)(1-c_S)^q + (1-p)^2(1-c_S)^q(1-(1-c_S)^q)\right] + (c_{\uparrow\downarrow} + c_{\downarrow\downarrow})\left[\frac{p^2}{2} + p(1-p)c_S^q + \frac{p}{2}(1-p)(1-c_S^q) + (1-p)^2(1-c_S^q)c_S^q\right], \tag{18}$$

$$\gamma^{-}_{\downarrow\uparrow} = c_{\downarrow\uparrow}\left[p + (1-p)(1-(1-c_S)^q) + \frac{p}{2}(1-p)(1-c_S)^q + (1-p)^2(1-c_S)^{2q}\right]. \tag{19}$$
