# Supplementary material for: Think then act or act then think?
Source: PLoS One. 2018 Nov 14;13(11):e0206166. doi: 10.1371/journal.pone.0206166 (PMC6235276; doi:10.1371/journal.pone.0206166)
Supplement: S3 Appendix — (PDF) [file pone.0206166.s003.pdf]

Think then act or act then think?

Arkadiusz Jędrzejewski, Grzegorz Marcjasz, Paul R. Nail, Katarzyna Sznajd-Weron

Supporting information: S3 Appendix.

Pearson correlation coefficient derivation.

Our starting point for deriving the explicit formula for the Pearson correlation coefficient $\rho_{S,\sigma}$ between states of public S and private σ opinions is its definition:

$$\rho_{S,\sigma} = \frac{\mathrm{Cov}[S,\sigma]}{\sqrt{\mathrm{Var}[S]\mathrm{Var}[\sigma]}}. \tag{1}$$

After applying the formulas for the covariance

$$\mathrm{Cov}[S,\sigma] = \mathrm{E}[S\sigma] - \mathrm{E}[S]\mathrm{E}[\sigma], \tag{2}$$

and the variances of both opinions

$$\mathrm{Var}[S] = \mathrm{E}[S^2] - \mathrm{E}[S]^2, \tag{3}$$

$$\mathrm{Var}[\sigma] = \mathrm{E}[\sigma^2] - \mathrm{E}[\sigma]^2, \tag{4}$$

the calculations boil down to finding all the above expected values. Both opinions can take one of two possible states 1 or -1. The probabilities of these events are equal to $\mathrm{P}(S=1) = c_S$ and $\mathrm{P}(S=-1) = 1 - c_S$, with analogical formulas for the private opinion σ. Having the outcomes of our opinions and their occurrence probabilities, we can easily calculate their expected values, for the public opinion we have:

$$\mathrm{E}[S] = \mathrm{P}(S=1) - \mathrm{P}(S=-1) = 2c_S - 1, \tag{5}$$

$$\mathrm{E}[S^2] = \mathrm{P}(S=1) + \mathrm{P}(S=-1) = 1. \tag{6}$$

Hence, after taking into account Eq. (3), the variance of S has the following form

$$\mathrm{Var}[S] = 1 - (2c_S - 1)^2 = 4c_S(1 - c_S). \tag{7}$$

Now, the last missing part is the expected value of the product of our two opinions $\mathrm{E}[S\sigma]$, but since S and σ are not independent, we have to use our four concentrations $c_{\uparrow\uparrow}$, $c_{\uparrow\downarrow}$, $c_{\downarrow\downarrow}$, and $c_{\downarrow\uparrow}$ that take into account the joint state of public and private opinions. Then we can write that $\mathrm{P}(S=1, \sigma=1) = c_{\uparrow\uparrow}$, and so on for the other state combinations. Having this, one can arrive at the following formula for the expected value of the product of public and private opinions:

$$\mathrm{E}[S\sigma] = c_{\uparrow\uparrow} - c_{\uparrow\downarrow} + c_{\downarrow\downarrow} - c_{\downarrow\uparrow}. \tag{8}$$

Using the fact that all the four concentrations must sum up to one $c_{\uparrow\uparrow} + c_{\uparrow\downarrow} + c_{\downarrow\downarrow} + c_{\downarrow\uparrow} = 1$ and the formula for the dissonance $d = c_{\uparrow\downarrow} + c_{\downarrow\uparrow}$, we can express Eq. (8) in terms of the dissonance alone:

$$\mathrm{E}[S\sigma] = 1 - 2d. \tag{9}$$

Finally, joining all the above results and using Eq. (1), we get the formula for the correlation coefficient presented in the main text

$$\rho_{S,\sigma} = \frac{c_S(1-c_\sigma) + c_\sigma(1-c_S) - d}{2\sqrt{c_S c_\sigma (1-c_S)(1-c_\sigma)}}. \tag{10}$$
